# Increasing Affinity of Interferon-**γ** Receptor 1 to Interferon-**γ** by Computer-Aided Design

**DOI:** 10.1155/2013/752514

**Published:** 2013-10-02

**Authors:** Pavel Mikulecký, Jiří Černý, Lada Biedermannová, Hana Petroková, Milan Kuchař, Jiří Vondrášek, Petr Malý, Peter Šebo, Bohdan Schneider

**Affiliations:** Institute of Biotechnology AS CR, v. v. i., Vídeňská 1083, 142 20 Prague, Czech Republic

## Abstract

We describe a computer-based protocol to design protein mutations increasing binding affinity between ligand and its receptor. The method was applied to mutate interferon-*γ* receptor 1 (IFN-*γ*-Rx) to increase its affinity to natural ligand IFN-*γ*, protein important for innate immunity. We analyzed all four available crystal structures of the IFN-*γ*-Rx/IFN-*γ* complex to identify 40 receptor residues forming the interface with IFN-*γ*. For these 40 residues, we performed computational mutation analysis by substituting each of the interface receptor residues by the remaining standard amino acids. The corresponding changes of the free energy were calculated by a protocol consisting of FoldX and molecular dynamics calculations. Based on the computed changes of the free energy and on sequence conservation criteria obtained by the analysis of 32 receptor sequences from 19 different species, we selected 14 receptor variants predicted to increase the receptor affinity to IFN-*γ*. These variants were expressed as recombinant proteins in *Escherichia coli*, and their affinities to IFN-*γ* were determined experimentally by surface plasmon resonance (SPR). The SPR measurements showed that the simple computational protocol succeeded in finding two receptor variants with affinity to IFN-*γ* increased about fivefold compared to the wild-type receptor.

## 1. Introduction

Recent developments in structural biology greatly enhanced our understanding of structural and energetic aspects of protein-protein interactions, and design of proteins with targeted modifications by rational, computer-aided techniques is becoming a standard tool of protein engineering [1–5]. Yet, full comprehension of affinity and specificity of these interactions remains a challenge, and reliable explanation, let alone prediction, of the intermolecular affinity solely by computational tools remains a difficult task. Difficulties to predict the actual outcome of the interactions between large protein molecules at the atomic level arise mainly from a large number of small contributions that are compensatory in nature. Their rigorous description from the principles of quantum mechanics is conceptually possible, but computationally intractable and empirical models of interactions suffer from inadequate description of certain types of interactions, namely, electrostatic, and complex types of processes, namely, hydration. The large size of modeled biological systems leads to incomplete sampling of the conformational space of the interacting molecules. Molecular dynamics and even relatively inexpensive techniques [6] are able to consider more complex changes of the polypeptide backbone; typically, scanned are only conformations of amino acid side chains, and changes of the polypeptide backbone are limited or not allowed altogether so that larger rearrangements of the interacting molecules are hard to predict. 

Despite all the limits in our understanding of the protein-protein interactions and technical obstacles related to their description, rational design of proteins with new or improved features is a promising alternative to experimental approaches for its speed and affordability [7]. The ingenious experimental techniques of directed evolution such as phage display [8–10] and ribosome display [11, 12] are able to generate proteins with new affinities and/or activities (“functions”). These techniques may completely change protein affinity from one binding partner to another and speed up mutational processes occurring in nature randomly. On the other hand, these experimental techniques shed little light on the interaction itself and therefore have a limited use for explaining why binding has changed. In contrast, computational methods that take into account the structures and energetics of the interacting molecules can provide rational insight into physical nature of the process of intermolecular recognition. Recently, emerging complex approaches to protein design combine methods of computational rational design and directed evolution [13–15] to benefit from both these techniques [16]. 

The present work included computer modeling, tools of molecular biology, and biophysical measurements into an accessible protocol to predict and test mutations increasing affinity of a model protein, IFN-*γ* receptor 1, to its binding partner, IFN-*γ*. IFN-*γ* is an important molecule of innate and adaptive immune responses in vertebrates [20–23]. Receptor 1 of IFN-*γ* is a part of the signal pathway of IFN-*γ* that binds to cellular receptor 1 and formation of the complex induces subsequent aggregation with distinct receptor 2; the ternary complex between IFN-*γ* and its two receptors then activates the JAK/STAT signaling pathway leading to establishment of immune response. The role of IFN-*γ* in immune system is used in diagnosis of tuberculosis. Stimulated production of IFN-*γ* by antigens present exclusively in infectious *Mycobacterium tuberculosis* is used in the so-called interferon-gamma release assays (IGRAs) to diagnose latent tuberculosis infection (LTBI). Commercial kits such as QuantiFERON-TB Gold or T-SPOT.*TB* achieve sensitive detection of stimulated levels of IFN-*γ* by reaction with specific antibodies in ELISA-like arrangement. Current increase of latent TB and emergence of highly resistant strains of *M. tuberculosis* inspired investigation of alternative approaches to the testing that would be based on molecular systems more robust than currently used antibodies. Our previous work [24, 25] has indicated that a small protein scaffold albumin-binding domain (ABD) of protein G from *Streptococcus* G148 [26] trained against its target by ribosome display [12] is one possible alternative. 

In this work, we decided to design high affinity IFN-*γ* binders based on a different protein molecule, the natural IFN-*γ* ligand, its receptor 1. Binding between IFN-*γ* and its receptor 1 occurring normally at the cellular membrane is also known to arise with the soluble extracellular portion of receptor 1 (hereafter labeled IFN-*γ*-Rx) [27, 28]. The existing crystal structures of the complexes [17, 29] and of the free human IFN-*γ* [30, 31] provided invaluable structural data to guide computational analysis. Mutations to modulate—increase as well as decrease—binding of IFN-*γ*-Rx to IFN-*γ* were searched for at the receptor residues forming the interface with IFN-*γ* and the interface receptor residues were subjected to computational mutational analysis by a modeling technique based on empirical force field. All 17 designed receptor mutants were then expressed, their affinities to IFN-*γ* were measured experimentally by surface plasmon resonance, and the predicted and the measured affinities were compared and discussed.

## 2. Materials and Methods

### 2.1. Selection of Amino Acid Mutations

**Figure 1:**
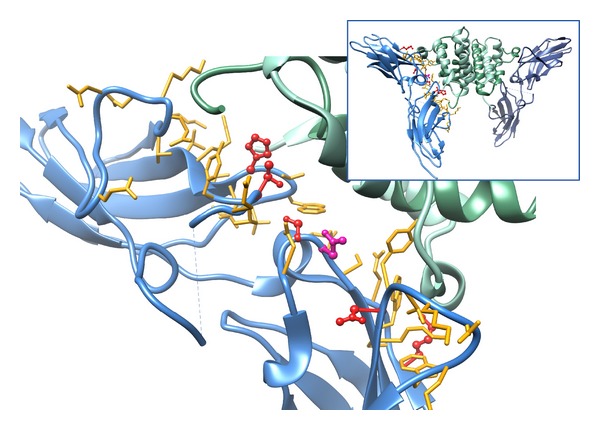
The interface between IFN-*γ* and the extracellular part of its receptor 1 (IFN-*γ*-Rx) from crystal structure 1fg9 [17]. Two IFN-*γ*-Rx molecules are drawn as blue cartoon and IFN-*γ* homodimer as green cartoon. The receptor residues forming the interface with IFN-*γ* are drawn as yellow sticks, the residues selected for mutations are highlighted in red, and the residue N96 in magenta. All the selected mutations are listed in Table 2.

The mutation analysis was based on analysis of the crystal structures of the complexes between the extracellular part of human interferon-*γ* receptor 1 (IFN-*γ*-Rx) and human IFN-*γ*, of PDB code 1fg9 [17] and 1fyh [29] contain five crystallographically independent molecules of IFN-*γ*-Rx in total; the asymmetric unit of 1fg9 contains three receptor molecules, but only two of them interact with IFN-*γ*; 1fyh has two receptor molecules interacting with IFN-*γ*. Therefore, there are four independent structures of the IFN-*γ*/IFN-*γ*-Rx complex. Potential mutations were searched for in the IFN-*γ*-Rx molecule, and the search was limited to its amino acid residues involved in direct interaction with IFN-*γ*. To make sure that all receptor residues potentially important for the interaction were included, we considered all residues within 6.0 Å from IFN-*γ* for mutations. A union of the four crystallographically unique interfaces consists of 40 receptor amino acid residues; they are depicted as wire models in Figure 1. The distances were calculated by the VMD program [32]. The variants potentially increasing the affinity of binding were selected by substituting the 40 residues of IFN-*γ*-Rx forming the interface with IFN-*γ* by the remaining 19 standard amino acid residues and calculating the changes of the interaction free energies, ΔΔ*G*. Mutations were calculated using the program FoldX (http://foldx.crg.es/) [33] independently for each of the four crystallographic interfaces, two from crystal structure 1fg9 and two from 1fyh. The crystal geometries were optimized and averaged by MD simulations independently for each interface. Two sets of calculations were run: the first set of ΔΔ*G* values estimated the influence of mutations on the stability of the whole IFN-*γ*/IFN-*γ*-Rx complex, the second evaluated change of the interaction between the receptor molecule and the rest of the IFN-*γ*/IFN-*γ*-Rx complex. The protocol for these computations is summarized in the Supplementary Text 1 available online at http://dx.doi.org/10.1155/2013/752514. 

### 2.2. Sequence Analysis

**Figure 2:**
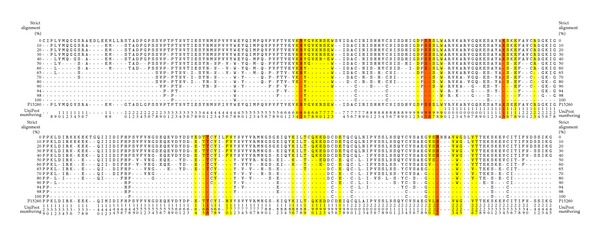
Conserved residues calculated by strict alignment of 32 sequences of the extracellular part of IFN-*γ* receptor 1 from 19 species. The receptor residues forming the interface with IFN-*γ* (i.e., residues no further than 6 Å from an IFN-*γ* atom) are highlighted in yellow; the residues selected for mutations are in red. All the selected mutations are listed in Table 2. Percentages of the conservation are shown on the left and right sides, sequence and numbering of UniProt P15260 on the bottom. Sequences used for the alignment are listed in Table S1. Numbering of the PDB entry 1fg9 can be derived from the UniProt one by subtracting 17. The alignment was computed by KAlign [18] as implemented in program Ugene [19].

The alignment was performed on 32 IFN-*γ* receptor sequences from 19 species: 12 sequences of primates (six human sequences, six from other primates), 15 sequences from other mammals, three from birds, one amphibian, and one viral (the viral protein is not a cellular receptor but highly specific IFN-*γ*-binding protein). The list of their GenBank codes is in Supplementary Table S1. The global sequence alignment was calculated using the KAlign [18] algorithm as implemented in the program Ugene (http://ugene.unipro.ru/, [19]); the resulting consensus sequence is shown in Figure 2. 

### 2.3. Molecular Dynamics (MD) of Wild-Type (WT) Complexes

MD simulations using the OpenMM [34] Zephyr [35] implementation of GPU accelerated version of GROMACS [36] suite of programs were used to test the stability, dynamic properties, and interaction free energies (Δ*G*) of the IFN-*γ*/IFN-*γ*-Rx complexes. The chains A, B, C, and D of the PDB structure 1fg9 and chains A, B, D, and E of the PDB structure 1fyh were used in the simulations. Missing residues were added using the Modeller suite of programs [37]; the pdb2gmx program using parameters provided by the Zephyr program determined ionization state. All MD simulations were performed using the following setup. Implicit solvation (GBSA, *ε* = 78.3, with collision interval of 10.99 fs) was used in combination with parm96 force field [38]. The initial IFN-*γ*/IFN-*γ*-Rx WT structure was optimized and the simulation was propagated at 300 K with time step of 2 fs. Snapshots of the geometry were saved every 10 ps throughout the simulation. 

In order to test the stability of various structural predictions, we performed several MD simulations of the WT as well as mutated IFN-*γ*/IFN-*γ*-Rx complexes. Three simulations of the WT complex consisted of a 100 ns MD run of the chains A, B, C, and D from 1fg9, which contain two interfaces, and two 20 ns runs for structure 1fyh, one for chains A, B and the other for chains D, E. These simulations demonstrated the stability of geometries of the crystal structures during the simulation. In the course of 100 ns 1fg9 simulation, instantaneous ΔΔ*G* values of one IFN-*γ*/IFN-*γ*-Rx interface switched to the value of the other interface and *vice versa*, suggesting sufficient sampling of the hypersurface of the free energy. For all the seventeen mutants, at least 10 ns MD simulations were run. They served as a reference for comparisons between calculated and measured affinities and to monitor the structural changes between the original crystal structures and the isolated solvated complexes. 

To check the theoretical stability of the mutated receptor molecules, 20 ns MD simulations of their complexes with IFN-*γ* were performed; simulations were conducted according to the same protocol as for the WT complexes. The interaction Δ*G*s of the complexes were recalculated using FoldX on 1,000 snapshot structures from the converged second half of each MD simulation. The resulting values were used for comparison with the experimentally determined dissociation constants of the mutants. 

### 2.4. Construction, Expression, and Purification of Recombinant Proteins

Codon-optimized synthetic open reading frame (ORF) encoding the residues 18 to 245 (P15260) of the extracellular domain of human IFN-*γ*-Rx was purchased from GenScript (Piscataway, NJ, USA). The ORF was cloned in frame as an *NcoI-XhoI* fragment into the pET-28b(+) vector (Novagen), resulting in the addition of N-terminal methionine (M*EMGT*) and C-terminal 6x His purification tag extension (*SIKG*LEHHHHHH). Residue mutations were introduced using the QuikChange II Site-Directed Mutagenesis Kit (Agilent Technologies) according to the manufacturer's protocol using the mutagenesis primers listed in Supplementary Table S2. All constructs were verified by DNA sequencing. 

**Figure 3:**
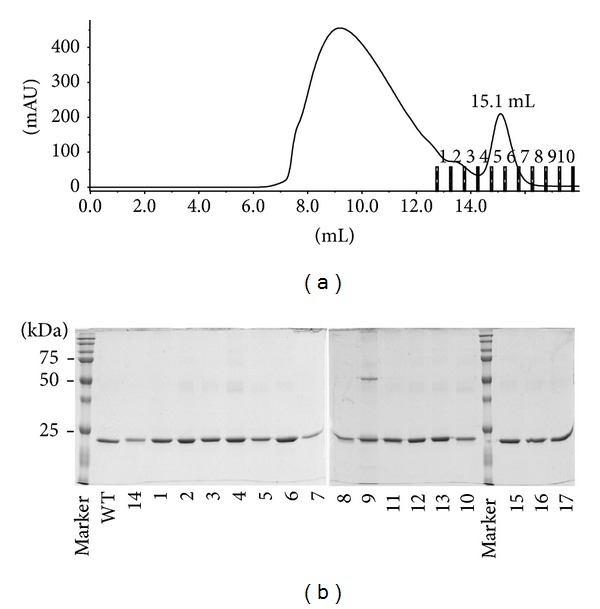
Purification of monomeric refolded recombinant 6xHis-tagged IFN-*γ*-Rx protein. (a) Typical chromatogram from separation of affinity-purified and refolded IFN-*γ*-Rx variants by gel permeation chromatography on Superdex 200 10/300 GL as described in Section 2. Fraction 6, containing the monomeric forms of refolded IFN-*γ*-Rx, was used for SPR measurements. (b) Analysis of purified soluble IFN-*γ*-Rx on 12.5% SDS-PAGE under nonreducing conditions. Proteins were extracted in 8 M urea from inclusion bodies and purified by metal affinity chromatography on Ni-NTA agarose as described in Section 2. Upon refolding by dialysis against urea-free buffer the monomeric fraction was separated as outlined above. IFN-*γ*-Rx with C-terminal His-Tag migrates at a molecular mass of 23 kDa when analyzed on non-reducing and at 27 kDa on reducing SDS-PAGE (not shown). Protein constructs are numbered as in Table 2.

The recombinant receptor proteins were produced in *Escherichia coli* BL21(*λ*DE3) (Novagen) at 37°C in LB medium containing 60 *μ*g/mL of kanamycin for 4 hours after induction by 1 mM IPTG. Cells were harvested by centrifugation (8,000 g, 10 min, 4°C), disrupted by ultrasound in 50 mM Tris buffer pH 8, and the protein was extracted from inclusion bodies in buffer A and affinity-purified close to homogeneity on Ni-NTA agarose (Qiagen). The receptor domain was eluted with 250 mM imidazole in buffer A (pH 8), refolded from urea by dialysis against 100 mM Tris-Cl pH 8, 150 mM NaCl, 2.5 mM EDTA, 0.5 mM cystamine, and 2.5 mM cysteamine overnight at 4°C. Monomeric refolded receptor protein was separated from aggregates and purified to homogeneity on a Superdex 200 10/300 GL (GE Healthcare) column run in PBS buffer pH 7.4 at 4°C (Figure 3). Monodispersity of the purified receptor protein was verified by dynamic light scattering (DLS) using Malvern Zetasizer Nano ZS90. 

Human natural IFN-*γ* is a homodimeric glycoprotein [39, 41], but glycosylation is dispensable for its biological activity [42]. Interferon-*γ* used in all analyses here was produced as a recombinant protein in the so-called single-chain form (IFN-*γ*-SC). The variant with the sequence taken from the previous report [31] was cloned in frame as an *NdeI-XhoI* fragment containing the stop codon into the pET-26b(+) vector (Novagen) and produced in *Escherichia coli* BL21(*λ*DE3). The cells were disrupted by ultrasound in buffer B (20 mM sodium phosphate buffer pH 7) and IFN-*γ*-SC was purified from the soluble cytoplasmic fraction on SP sepharose HP (GE Healthcare) equilibrated in buffer B using a linear gradient of NaCl. 

### 2.5. Measurement of the Thermal Stabilities of the Mutants and WT

Protein melting temperature (Tm) was determined by fluorescence-based thermal shift assay (TSA) using fluoroprobe SYPRO Orange dye (Sigma Aldrich). The TSA was performed in “CFX96 Touch Real-Time PCR Detection System” (Bio-Rad) using FRET Scan Mode. The final volume of assay was 25 *μ*L, concentrations of IFN-*γ*-R variants 3 *μ*M, and dye at 8-fold dilution from 5000-fold stock. The reference was dye in assay buffer (PBS buffer pH 7.4) without protein. Samples in capped “Low Tube Strips, CLR” (Bio-Rad) were spun down immediately before the assay to remove possible air bubbles. For thermal denaturation, the samples were heated from 20°C to 75°C with stepwise increment of 0.5°C per minute and a 30 s hold step for every point, followed by the fluorescence reading. Reference subtracted data were normalized and used for first derivative calculation to estimate the melting temperature. 

### 2.6. Measurement of the Interaction between IFN-*γ* and Its Receptor

Interactions between IFN-*γ*-Rx variants and IFN-*γ*-SC were measured by the technique of surface plasmon resonance (SPR) using the “ProteOn XPR36” instrument (Bio-Rad) on a HTG sensor chip with surface activated with Ni^2+^ cations (10 mM NiSO_4_, 10 mM MES pH 6). His-tagged receptor molecules were diluted to concentration 10 *μ*g/mL in PBST running buffer (PBS pH 7.4, 0.005% Tween20) and immobilized at a flow rate of 30 *μ*L/min for 60 s. Purified IFN-*γ*-SC was diluted in PBST running buffer to concentrations ranging from 1.2 to 99 nM and passed over the sensor chip. Association of IFN-*γ*-SC with receptors was adjusted to 90 seconds at a flow rate of 100 *μ*L/min and dissociation occurred in PBST running buffer for 10 min at the same flow rate. His-tagged Fe-regulated protein D (FrpD) from *Neisseria meningitidis* [43] was used as a negative control in the reference channel. The signal was corrected for nonspecific binding of the protein to the chip surface by subtraction of the response measured on uncoated interspots and in the reference channel. The doubly referenced data were analyzed and fitted to the 1 : 1 “Langmuir with drift” binding model using ProteOn Manager version 3.1.0.6 software. Regeneration of the HTG sensor chip was accomplished using 300 mM EDTA pH 8.5. Reported SPR affinities were measured at 25°C. 

## 3. Results and Discussion

### 3.1. Analysis of the Crystal Structures

The mutation analysis IFN-*γ*-Rx was limited to 40 amino acid residues that were identified as closer to 6.0 Å from IFN-*γ* in the crystal structures of IFN-*γ*/IFN-*γ*-R1 complexes (PDB codes 1fg9 [17] and 1fyh [29]).  Table 1 compares root mean square deviations (rmsd) between the main chain atoms of these 40 residues at the interface and 40 randomly selected residues outside the interface and shows that all four IFN-*γ*-Rx molecules are quite similar: the residues involved in direct interaction with IFN-*γ* deviate from the reference chain D of 1fg9 by less than 0.5 Å, residues outside the interface by less than 2 Å. Notably, the structure of the receptor molecule, which is not in direct interaction with IFN-*γ* (chain E in 1fg9), differs from the other receptor molecules by more than 4 Å, significantly more than they differ from each other. Therefore, recognition between IFN-*γ* and its receptor 1 is likely to narrow conformational space available for the receptor molecule, a feature advantageous for the modeling effort. 

### 3.2. *In Silico* Design of Mutants

To identify mutations increasing the affinity of IFN-*γ*-Rx to IFN-*γ*, we replaced each of the 40 receptor interface residues by the remaining 19 natural amino acids and calculated two types of changes of free energy (ΔΔ*G*) using the web-based program FoldX [33]. First, we estimated the stability of the mutated receptor by calculating ΔΔ*G* in the complex. These ΔΔ*G* values estimate the stability of the receptor molecules. Next, we tested how the receptor mutations change binding to IFN-*γ* and these ΔΔ*G* gauge the change of affinity. Two example matrices of ΔΔ*G* values are in Supplementary Table S3. Because the calculated ΔΔ*G* values may differ between the four crystallographic interfaces, both types of the interacting matrices were independently calculated for all four interfaces. The differences between the corresponding ΔΔ*G* values in the four stability and four affinity matrices are however not large because the four receptor molecules interacting with IFN-*γ* are structurally similar (Table 1). 

Favorable (i.e., negative) stability and affinity ΔΔ*G* values calculated for all four interfaces indicated promising mutations. This energy-based criterion for selection of mutants was supplemented by considering conservation of the receptor sequences in various species to avoid mutating the most preserved residues that may carry significant structural or functional role. Residues that were identified as conserved in more than 65% of 32 IFN-*γ* receptor sequences from 19 species (Figure 2, Supplementary Table S1) were not considered for mutations. By combining the criteria of energy stabilization and sequence variability, we selected nine most promising mutations (Table 2). 

In addition to these nine single amino acid mutations, we decided to evaluate the additive effects of introduction of multiple mutants. Therefore, three mutations, N70G, S95R, and H222R, which were predicted to stabilize the interface significantly and are distant from each other, were combined into one triple and three double mutants so that all seven possible mutual combinations of the three mutations were studied. These selected mutants are schematically depicted in Figures 1 and 2 and listed in Table 2 under numbers 1–14. 

All but one receptor constructs were designed prior to any experimental determination of their affinities. The only “second-generation” variant is the double mutant N96W + H222R (number 10 in Table 2) that was expressed because the single mutant N96W had a high experimental affinity and H222R showed neutral binding behavior, while these two single mutations are sequentially distant so that we assumed that they might influence each other the least. 

### 3.3. Experimental Determination of the Affinities of the Mutants

All mutants proposed for construction (Table 2) were expressed, purified, and refolded making use of the protocol developed for the wild-type (WT) IFN-*γ*-Rx as described in Section 2. Affinities of WT and all mutants to a single-chain variant of IFN-*γ* (IFN-*γ*-SC, see Section 2 were measured by SPR. The SPR data are summarized in Table 3. 

The mutants can be qualitatively divided into three groups. First, those that have higher affinity (lower *K*_*d*_ values) compared to the WT receptor, second, mutants with affinity close to that of WT, and third, mutants with affinity lower than WT (higher *K*_*d*_ values). A significant, about five-fold, increase of affinity compared to WT was observed for two mutants: N96W and N96W + H222R. A large group of mutants have their *K*_*d*_ values close to those of WT, for example, mutants N70G, N96F, and the triple mutant. From the formal statistical point of view, some of these *K*_*d*_ values may be significantly different from the values for WT, but the biological relevance of these changes is negligible. Finally, a few mutants, for example, N65R, S95R, or T166Y, have their affinities about two to three times lower than that of WT. 

To test whether sequentially and spatially distant mutations affect the binding to IFN-*γ*-SC independently or in accord, three single mutations, N70G, S95R, and H222R, which were about 25 amino acids apart in sequence and more than 20 Å apart in 3D space, were combined to produce three double and one triple mutants. The cooperativity of mutations was checked by comparing the changes of experimental binding affinities (ΔΔ*G*) for the seven mutants in the series. Data in Table 2 show that experimental values of ΔΔ*G* of the double mutants are approximately the sum of contributions from the single mutants and ΔΔ*G* of the triple mutant is the sum of the values for the three single mutants. In general, the interplay of multiple mutations cannot be ruled out as nonadditive energetic effects have been observed for mutations at positions separated by more than 9 Å [44]. In that study, association and dissociation rates have had the opposite effects on the overall nonadditivity of the mutants: association has been responsible for the cooperativity, while dissociation for the anticooperativity (less-than-additive energetics). 

To monitor nonrandomness of predictions to *increase* the receptor affinity to IFN-*γ*, we selected a smaller set of variants that were predicted to *lower* the receptor affinity. To find these mutants, we searched for ΔΔ*G* lowering the affinity but still increasing the stability of the receptor molecule itself. Three selected mutants are listed in Table 2 under numbers 15–17. The dissociation constants of mutants H222D and S71E are about two times lower than *K*_*d*_ of WT (2.2 and 2.0 times, resp.); the third mutant, Y66L, has about the same affinity as WT. These experimental *K*_*d*_ values thus support the general applicability of the computer predictions and the ability of our computer modeling protocol to suggest mutations that lead to the desired effects, be it affinity increase or decrease.

Our best single mutant (N96W) increases the binding free energy by about 5 kJ/mol; the corresponding decrease of *K*_*d*_ is about fivefold; binding improvement is generally comparable to other studies. A recent study has enhanced affinity of an antibody fragment to the I-domain of the integrin VLA1 [45] by about an order of magnitude by mutating four residues at the antibody part of the interface. Similarly, five amino acid substitutions increased affinity between integrin antigen LFA-1 and its ligand about twentyfold [46]. Single amino acid substitutions in decoy receptor TLR4 constructed of leucine-rich repeats increased affinity to myeloid differentiation protein 2 about tenfold [47]. Interestingly, this study reports high cooperativity among the single mutations as the affinity of double mutants has been reported up to a thousand-times higher compared to WT. Computer model of binding between acetylcholine esterase and its inhibitor fasciculin [48] has predicted that increase of affinity can be achieved by mutating five interface fasciculin residues. However, to achieve a better binding, at least one of the five mutations had to be scrapped and actually the tightest interaction (sevenfold increase) occurred with just one of the originally designed mutations. Using the same software, ORBIT, binding between peptides derived from myosin light chain kinase and calmodulin was modeled [49], and similarly to the previously mentioned study, some predicted mutations led to increase but others to decrease of affinity. 

Despite the complicated nature of protein-protein interactions, a few general rules have been drawn from these and other studies: polar residues replacing hydrophobic ones destabilize complex formation and replacement of charged by hydrophobic residues increases binding [50]. 

Considering that the presented protocol was based on a straightforward geometric analysis of the crystal interface and the changes of the interaction free energy were estimated by an empirical force field containing many simplifications, a decrease of affinity in about a half of mutants designed to increase the affinity is not surprising, especially in the light of simplicity of the computational methods and complexity of the system. In our opinion, a drawback of the computer-driven rational design based on energy calculations stems from a different fact than is the ratio between true and false positives: since many predictions of stabilizing mutations are incorrect, we should expect not only false positive but also the false negative error (type II error), ΔΔ*G* values of stabilizing mutations calculated incorrectly as destabilizing. Because false negative predictions are never tested, computer predictions may miss mutations that would stabilize the complex more than any of the actually selected and tested mutants. This disadvantage does not exist in experimental protocols, such as ribosome display, that not only scan an incomparably larger portion of the overall sequence space, but also scan it without any prejudice. Regardless of its limits, *in silico* design of mutations increasing affinity between ligand and its receptor can be a useful tool because ligand-receptor interactions do not evolve for the maximal affinity but for affinity optimal to enable proper signalization. It is therefore likely that interfaces of most ligand-receptor complexes can be modified to increase their affinity. 

### 3.4. Biochemical and Statistical Significance of the SPR Data

SPR measurements for WT and most receptor variants were repeated to test reproducibility (or rather repeatability) of the data. The data listed in Table 3 were measured on three different SPR chips and anchored receptor molecules originated from different batches but always using one batch of IFN-*γ*-SC. Measurements under these conditions are reliable and sufficiently accurate as is demonstrated by estimated standard deviations of the *K*_*d*_ values between 4 and 9%. The formal statistical significance of the differences between *K*_*d*_ values of the mutants and WT is given in Supplementary Text 2. The average value of *K*_*d*_ of WT receptor was determined as 30.8 ± 0.9 nM in our SPR experiments (Table 3). This value agrees well with the literary value 27 ± 9 nM determined by isothermal titration calorimetry for interaction between recombinant IFN-*γ*-SC and IFN-*γ*-Rx also at 25°C but in a different buffer (10 mM Pipes at pH 7.1, 150 mM NaCl) [31]. 

Dissociation constants of two variants can be used to calculate the changes of Gibbs energy of their interaction; for dissociation constants of WT (*K*_*d*_)_WT_ and a mutant (*K*_*d*_)_mut_:

(1) ΔΔG=−RT ln⁡(Kd)mut−{−RT ln⁡(Kd)WT}=−RT ln⁡{(Kd)WT(Kd)mut}.

The experimental ΔΔ*G* values in Table 2 were calculated from *K*_*d*_ values measured using the same batch of IFN-*γ*-SC for each particular pair of WT and a mutant (see Supplementary Text 2). When the ΔΔ*G* values were calculated from measurements using four different batches of IFN-*γ*-SC (but always the same batch for WT and a mutant), their mean values agreed with the values of the single-batch measurement but the uncertainty limits grew. Direct comparison of ΔΔ*G* values obtained from measurements using different batches of IFN-*γ*-SC is thus less reliable. For two mutants N70G and N96W, the average ΔΔ*G* and uncertainty limits were −0.7 ± 0.7 and −3.7 ± 1.0 kJ/mol, respectively. 

### 3.5. Kinetics and Equilibrium of Binding

**Figure 4:**
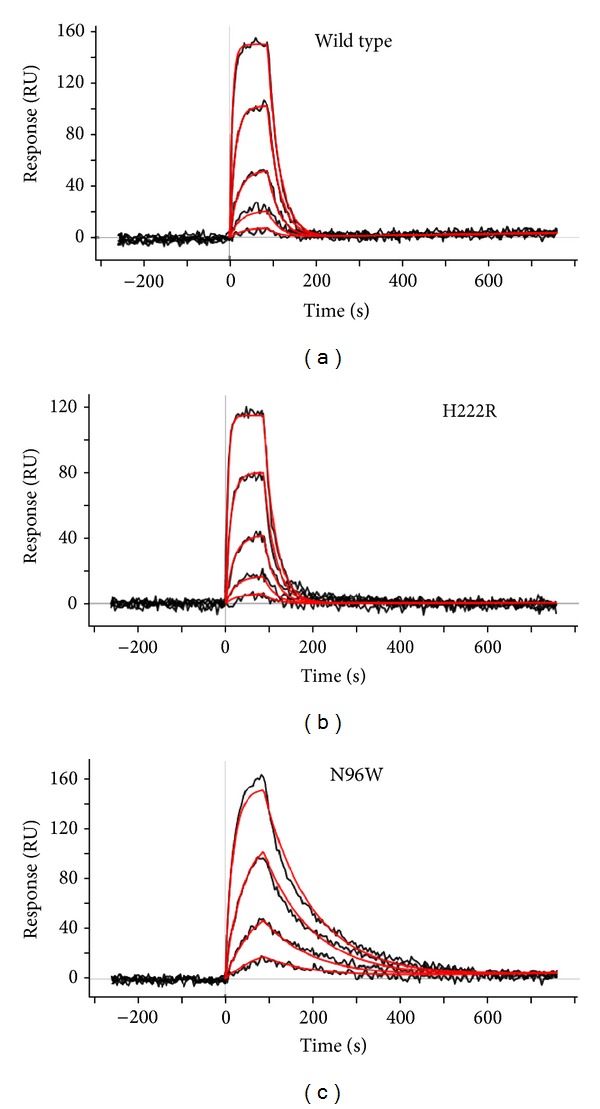
Association and dissociation curves of the SPR experiments. Most IFN-*γ*-Rx variants behave similarly as the wild-type (a) and mutant H222R (b): they bind IFN-*γ*-SC very fast but also release it fast. Two high-affinity binders, mutant N96W (c) and the double mutant N96W+H222R, bind the IFN-*γ*-SC molecules for a longer time, thus increasing the affinity to IFN-*γ*. The SPR experimental signal is in black; the fitted curves from which the association and dissociation kinetic constants are calculated are in red. The SPR data for all variants are in Table 3.

Table 3 shows that the mutants associate with IFN-*γ*-SC with similar kinetics (measured by association rate constant, *k*_*a*_) but for most, the fast association is followed by fast dissociation (dissociation rate constant, *k*_*d*_). However, the two mutants with significantly increased affinity to IFN-*γ*-SC, N96W, and N96W + H222R, dissociate much more slowly. Their kinetic behavior distinguishes them from the other mutants as illustrated in Figure 4, which compares the SPR interaction curves of two receptor mutants exhibiting fast release and one of the high-affinity mutants N96W with much slower release of IFN-*γ*-SC. Considering the formula to calculate dissociation equilibrium constant, *K*_*d*_ = *k*_*d*_/*k*_*a*_, the slower off-rates of mutants N96W and N96W + H222R, that is, smaller values of *k*_*d*_, explain a large part of the increase of their higher affinity to IFN-*γ*-SC (lower values of *K*_*d*_). The process of dissociation distinguishes these two mutants from the other receptor constructs and they are thus interesting not only for their thermodynamic properties, affinity, but also for the different kinetic characteristics of the interaction. In this context, the ability of SPR technique to determine kinetics of binding is crucial. The potential of this technique has been used to explain affinity between 14 mutations of an antibody and lysozyme [51] and to provide information about chemical aspects of this interaction. 

Both alternate strategies for affinity increase, one based on faster binding, the other on a slower release of the complexed molecules, have been reported. Clark et al. [45] and this study reported increased affinity caused by a slower dissociation; other studies [52–55] have reported that the affinity increase of mutants is caused by higher rates of association rather than slower dissociation. Faster association has been attributed to increased electrostatic attraction between the binding partners, for example, for binding between TEM1 beta-lactamase and its protein inhibitor BLIP [53], but it can also originate from mutations of noninterfacial residues as in study [54]. Optimization of electrostatic contributions for protein-protein interactions has been recently reviewed [56]. 

The importance of kinetic effects in forming IFN-*γ*/IFN-*γ*-Rx complexes is indirectly supported by failure of PISA [57] to recognize the biologically “correct” complexes in both 1fg9 and 1fyh. PISA is a computer method estimating the stability of macromolecular interfaces from their crystal structures. It should be stressed that it is generally highly successful in discerning interactions stable in solution from “nonspecific” crystal-forming interfaces but in case of IFN-*γ* complexes, PISA recognizes correctly biological unit of only one, that formed with viral IFN-*γ* binding protein, PDB code 3bes [58]. In this case, rigidity of the receptor-like molecule and avidity of the interaction strengthen the binding. Stiffening of the interacting molecule(s) may be an alternative search strategy for high-affinity mutants as has been convincingly illustrated by the design of a “superkine” protein molecule [59]. 

### 3.6. Structure and Binding

Design of high-affinity mutants by computer-driven design relies on structural information. The knowledge of experimental structure even at a relatively low crystallographic resolution around 3 Å provides firm constraints for search of energetically favorable replacements and removes unavoidable uncertainty of computer-predicted structural models. Structural variations between crystal structures allow estimating the extent of flexibility of the molecules. The small differences between the four crystallographically independent structures of the IFN-*γ*/IFN-*γ*-Rx complex observed in structures 1fg9 [17] and 1fyh [29] (Table 1) indicate that the structural variations can be expected to be relatively small and that the energy computations, which are sensitive to structural variances, may be expected to provide reliable estimates. Structure of the third receptor molecule observed in structure 1fg9 that is not considered to be biologically relevant [17] differs from the structures of two complexed receptors. 

The structural explanation of the increased affinity of the two high-affinity variants N96W and N96W + H222R is not straightforward. Based on snapshots from the MD simulations, the replacement of asparagine by tryptophan does not generate easily identifiable interactions such as hydrogen bonds or stacking between the tryptophan aromatic ring of its –N(H)– group and the rest of the receptor molecule or nearby atoms of IFN-*γ*-SC. On the contrary, one H-bond present in the WT complex is actually weakened. Surprisingly, instead of the expected stiffening of the nearby groups, the bulky tryptophan increased the mobility of several receptor residues, namely, N65 and Y66; values of their root mean square fluctuation grew by a factor of three. The only obvious stabilizing effect of a large tryptophan residue compared to a smaller asparagine is a larger number of van der Waals contacts it forms. We hypothesize that the higher stability of the complex, namely, its longer dissociation compared to WT, is driven by the entropic destabilization of a large tryptophan residue when it is exposed to the aqueous environment. The hydrophobic destabilization at the position 96 and the related increased flexibility of the receptor molecule suggested by the MD simulations for the N96W mutants help to rationalize their measured lower melting temperatures compared to WT. They were estimated to be 55°C for IFN-*γ*-Rx WT, 48°C for N96W, and 47°C for N96W + H222R (curves of thermal stabilities are in Supplementary Figure S1). The increase of flexibility at the interface of the complex suggests that ignoring entropic contribution to free energy [60] is not a generally acceptable approach. Better understanding of the stabilization effect of the tryptophan at the receptor position 96 clearly requires further study, at least reliably characterized temperature dependency of *K*_*d*_ values; our data acquired using receptor molecules anchored on the Ni^2+^-coated HTG SPR chip (Supplementary Table S4) serve as initial estimates of the full thermodynamic description of the IFN-*γ*-SC/IFN-*γ*-Rx system. 

### 3.7. Comparison between Computer-Predicted and Experimental Affinities

Table 2 summarizes the values of changes of binding free energies, ΔΔ*G* from the initial FoldX calculations (used to make predictions), averages from the FoldX calculations on a thousand MD snapshots, and from the SPR measurements. The most noticeable difference between the calculated and experimental values seems to be the scale: computer predictions clearly overestimated the magnitude of ΔΔ*G*s. Comparison between ΔΔ*G* values calculated by FoldX for the initial structure and the values averaged over the MD snapshots indicated large differences; the values shifted for example, from −5 to +17 for N65R or from −13 to −0.6 for N96F. 

After comparing FoldX calculated values of ΔΔ*G* at the four crystallographic interfaces, we observed large fluctuations in several components of the FoldX force field, especially in the solvation and electrostatic contributions (data not shown). Considering relatively small structural variations between the individual crystal interfaces, we suggest that the Achilles heel of the predictions is a limited accuracy of modeling of solvation effects, the equilibrium between charged and uncharged states, and the contribution of polarizability of large ionized particles including amino acid residues. The computations are also likely underestimating (or systematically neglecting) possible mutation-induced rearrangements of the backbone. FoldX potential is likely to overestimate interaction energy of charged arginine as it repeatedly suggested to mutate different residues to R (N65R, S95R, and H222R). All these suggested mutations to arginine are incorrect in the light of the experimental results regardless whether they were predicted as stabilizing or destabilizing by the MD calculations. However, the overall performance of the FoldX force field was satisfactory especially in the light of a recent report that no empirical potential predicts correctly all types of interaction [61]. 

Perhaps surprisingly, ΔΔ*G* values based on the 10–20 ns MD simulations, which are sufficient to rearrange the backbone, did not offer any systematic improvement of the agreement with the experimental ΔΔ*G* values over the FoldX predictions on either the crystal structure or on the averaged MD structures. Computationally more demanding MD data shifted the ΔΔ*G* values in both directions, closer to the experimental values as in the case of N65R, S95R, and N96F, but also off them, as for mutants of K115Y and H222R. Despite the currently prevailing opinion that long MD simulations are indispensible for reliable description of molecular systems and prediction of affinity modulation [62], we conclude that for the purpose of mutant design, predictions based on simply relaxed crystal structure can be as reliable as predictions based on much more laborious and expensive calculations. Also other authors [60] have observed that full MD simulation is not more successful in prediction of mutants than simpler approaches, and that the inclusion of nonlocal flexibility of the to-be mutated protein structure led to a higher number of false positive predictions [45]. 

## 4. Conclusions

In the present work, we used a generally applicable computer protocol to identify mutations increasing binding of two proteins and applied it to increase affinity of the extracellular domain of human interferon-*γ* receptor 1 (IFN-*γ*-Rx) to its natural ligand IFN-*γ*. The best mutant had affinity five times larger than the wild-type receptor. The computer-aided protocol was based on analysis of available crystal structures 1fg9 [17] and 1fyh [29], consideration of sequence conservation among 32 receptor sequences from 19 species, and free energy calculations by a web-based empirical force field FoldX [33]. We designed nine single-site mutants, five double, and one triple mutant. All these mutants were expressed as recombinant proteins in *Escherichia coli*, purified, and refolded, and their affinities to recombinant IFN-*γ* were measured by surface plasmon resonance (SPR) with IFN-*γ* as analyte and IFN-*γ*-Rx anchored to the surface of the chip. Table 3 shows that of nine single mutants selected for the analysis, one, N96W, exhibits about fivefold increase of affinity to IFN-*γ* compared to WT receptor (the corresponding ΔΔ*G* is −5 kJ/mol). In addition, one double mutant combining two single mutations (N96W + H222R) showed a similar increase of affinity, likely brought about also by the N96W mutation. The higher affinity of the variants containing the N96W mutation was a consequence of their slower rate of dissociation (off-rates) than that observed for WT, the association rates (on-rates) of WT and all mutants were about the same. 

The results demonstrate that computer-aided design of single-site amino acid mutations is an applicable strategy to increase binding between two complex proteins with already highly optimized interface and affinity in the nanomolar range. 

## Figures and Tables

**Table 1:** Structural similarity of the IFN-*γ* receptor molecules (IFN-*γ*-Rx) at and outside the interface with IFN-*γ*. Four receptor chains from crystal structures 1fg9 [17] and 1fyh [29] are compared to receptor chain D of 1fg9.

PDB	rmsd (Å)^a^	rmsd (Å)^b^
Code:chain	40 interface residues	40 random residues
1fg9:C	0.60	1.66
1fg9:E	4.16	4.32
1fyh:B	0.58	1.42
1fyh:E	0.59	1.06

^a^Root mean square deviations (rmsd) between the four IFN-*γ*-Rx molecules (labeled PDB_ID:chain) and the chain D of 1fg9. Deviations are calculated between the positions of the main chain atoms of the 40 residues forming the interface with IFN-*γ*.

^b^Root mean square deviations (rmsd) between the four IFN-*γ*-Rx molecules (labeled PDB_ID:chain) and the chain D of 1fg9. Deviations are calculated between the positions of the main chain atoms randomly selected outside the 40 residues forming the interface with IFN-*γ*.

**Table 2:** Calculated and experimental values of the changes of free energy, ΔΔ*G*, of the interaction between IFN-*γ*-Rx mutants and IFN-*γ*-SC relative to the wild-type receptor.

Construct		The best ΔΔ*G* ^c^	ΔΔ*G* from MD^d^	Experimental ΔΔ*G* ^e^	esd^f^
ID^a^	Mutation^b^	(kJ/mol)	(kJ/mol)	(kJ/mol)	(kJ/mol)
1	N65R	−5.4	17.3	2.1	—
2	N70G	−5.4	0.3	−0.6	—
3	S95R	−8.3	11.8	2.1	—
4	N96F	−13.0	−0.6	−0.2	—
5	N96W	−9.9	−6.1	−3.9	0.2
6	K115Y	−0.3	−9.6	0.7	—
7	T166M	−5.8	−5.4	2.0	—
8	T166Y	−9.8	0.9	2.5	—
9	H222R	−6.9	−15.8	−0.1	0.2
10	N96W + H222R	−7.1	−7.1	−5.0	0.2
11	N70G + S95R	−7.3	2.7	1.5	—
12	N70G + H222R	−4.6	−7.3	−0.3	—
13	S95R + H222R	−11.4	−10.8	1.5	—
14	N70G + S95R + H222R	−15.8	−5.6	0.5	0.1
15	Y66L	2.1	11.8	0.0	—
16	S71E	9.6	19.6	1.6	—
17	H222D	6.7	5.8	2.0	—

^a^Mutants 1–14 are single, double, and triple mutants designed to increase affinity to IFN-*γ* compared to WT. Mutants 15–17 were designed to lower the affinity between IFN-*γ* and IFN-*γ*-Rx but not to destabilize the unbound IFN-*γ*-Rx.

^b^Residues are numbered as in the UniProt entry P15260.

^c^For mutants 1–14, the most negative (most stabilizing) values obtained at the four crystal interfaces by FoldX [33]. For mutants 15–17, the ΔΔ*G* listed are for the least positive (least destabilizing) interface.

^d^Averaged ΔΔ*G* values calculated by FoldX on structures taken from snapshots of 10 to 20 ns MD runs by GROMACS [36].

^e^ΔΔG values determined from experimental SPR values of dissociation equilibrium constants *K*_*d*_ as ΔΔ*G* = −RTln⁡{(*K*_*d*_)_WT_/(*K*_*d*_)_mut_}.

^f^Estimated standard deviations for the experimental values of ΔΔ*G* with the number of independent SPR measurements *N* > 2 (Table 3).

**Table 3:** Affinity between IFN-*γ*-SC and IFN-*γ*-Rx mutants was predicted to increase affinity measured by surface plasmon resonance (SPR).

Construct		*k*_*a*_∗10^−6^	*k*_*d*_∗10^2^	*K*_*d*_	*N* ^e^	esd (*K*_*d*_)
ID	Mutation^a^	(1/Ms)^b^	(1/s)^c^	(nM)^d^		(nM)^f^
WT	—	1.24	3.78	30.8	14	1.5
1	N65R	0.882	6.28	71.2	1	na
2	N70G	1.12	2.64	23.6	1	na
3	S95R	0.650	4.54	69.8	2	na
4	N96F	1.01	2.83	28.0	1	na
5	N96W	1.43	0.909	6.34	4	0.49
6	K115Y	0.979	3.91	39.9	1	na
7	T166M	0.933	6.39	68.5	1	na
8	T166Y	0.940	7.82	83.1	1	na
9	H222R	1.19	3.49	29.4	6	1.9
10	N96W + H222R	2.40	1.00	4.16	3	0.37
11	N70G + S95R	0.889	4.94	55.9	2	na
12	N70G + H222R	1.46	3.91	26.9	2	na
13	S95R + H222R	1.05	5.90	56.3	2	na
14	N70G + S95R + H222R	1.09	4.01	37.0	5	2.1

^a^Residues are numbered as in UniProt P15260.

^b^Kinetic constant of association, *k*_*a*_.

^c^Kinetic constant of dissociation, *k*_*d*_.

^d^Dissociation equilibrium constants *K*_*d*_ calculated as *k*_*d*_/*k*_*a*_.

^e^Number of independent SPR measurements.

^f^Values of the estimated standard deviation (esd) of *K*_*d*_ are shown for mutants with three and more measurements (listed in column *N*).

Confidence limits calculated from the Students *t*-distribution at the 95% level are ±0.85, ±0.78, ±1.9, ±0.93, and ±2.6 nM for WT, N96W, H222R, N96W + H222R, and N70G + S95R + H222R, respectively.
