# Supplementary material for: Increasing Affinity of Interferon-γ Receptor 1 to Interferon-γ by Computer-Aided Design
Source: Biomed Res Int. 2013 Oct 2;2013:752514. doi: 10.1155/2013/752514 (PMC3807708; doi:10.1155/2013/752514)
Supplement: Supplementary file 1 [file 752514.f1.pdf]

Supplementary material

Increasing affinity of interferon-γ receptor 1 to interferon-γ by computer-aided design

Pavel Mikulecký, Jiří Černý, Lada Biedermannová, Hana Petroková, Milan Kuchař, Jiří Vondrášek, Petr Malý, Peter Šebo, and Bohdan Schneider (*)

Institute of Biotechnology AS CR, v. v. i., Vídeňská 1083, CZ-142 20 Prague, Czech Republic

(*) Corresponding author:
phone: +420 241 063 624 ; fax: +420 296 443 610
email: bohdan@img.cas.cz ; http: http://www.structbio.eu/BS/

Supplementary text 1. Methods

Protocol of the computations.

The necessary software was obtained free of charge from following web addresses:

1. Modeller 9.12, http://salilab.org/modeller
2. FoldX 3.0 Beta 4, http://foldx.crg.es
3. OpenMM Zephyr 2.0.3, https://simtk.org/home/zephyr (contains GPU accelerated version of GROMACS)

1. Modeller suite of programs version 9.12 was used to model residues missing ("not visible in the electron density") from the 1fg9 crystal structure.

The following loop and C-terminal residues were added (residue numbers according to the PDB):

VAL C 142, ASP C 143, TYR C 144, VAL D 142, ASP D 143, TYR D 144, ASP D 145, PRO D 146, GLU D 147

PHE D 222, ASN D 223, SER D 224

The missing residues were constructed employing the "loopmodel" routine and fast MD refinement (by "`refine.fast`"). The lowest energy structure was chosen from ten models and used for the subsequent mutation analysis. All missing residues were outside the interface area.

2. The *in silico* mutation of selected interface residues was performed using locally installed binary of the FoldX program.

The analyzed coordinates included four chains from each crystal structure – ABCD from 1fg9, and ABDE from 1fyh, respectively. The structures of 20 mutants at all forty mutated positions (see Figure 2) in PDB format were generated using the

```
<PositionScan>#,ONELETCHAINRESNR
```

keyword, where the `ONELET`, `CHAIN`, and `RESNR` were replaced for each interface residue by its one letter code, PDB chain, and the residue number.

The FoldX program calculated simultaneously also the ΔΔG values of these mutations. These ΔΔG values measured the effect of mutations to the overall stability of the IFN-γ/IFN-γ-Rx complex.

To address the (de)stabilizing effect of interface mutations on the receptor binding, the FoldX keyword

```
<AnalyseComplex>#,CHAIN
```

was used, with the CHAIN representing the IFN-γ-Rx chain. This keyword performs analysis of the interaction of the selected chain with the rest of the structure, for instance interaction between chain C and the remaining chains ABD in 1fg9.

3. The graphical interface OpenMM Zephyr was used for preparation and execution of the MD simulations.

This graphical user interface (GUI) is shielding a potential user from the detailed setup of MD simulation. The preparation of successful simulation contains choosing the starting PDB structure from within the GUI, selecting the desired combination of force field, other conditions, e.g. solvation model and temperature, and clicking the Simulate button. We used the default

parameters, parm96 force field, implicit solvation (GBSA, ε = 78.3, with collision interval of 10.99 fs), temperature of 300 K, and time step of 2 fs. OpenMM Zephyr automatically runs the GROMACS (sub)programs with the proper parameters. The protocol includes:

Transformation of PDB file PROTEIN.pdb to PROTEIN_processed.pdb with `ffamber` naming conventions.

Running `pdb2gmx` to protonate and set the force field parameters:

```
pdb2gmx -f PROTEIN_processed.pdb -o PROTEIN_processed.gro -p
PROTEIN_processed.top -ter -ignh
```

Running `editconf` to define periodic solvation box:

```
editconf -bt cubic -d 0.7 -f PROTEIN_processed.gro -o
PROTEIN_processed.box.gro
```

Running `grompp` and `mdrun_openmm`: run first for restrained optimization of hydrogen atoms:

```
grompp -f em.mdp -c PROTEIN_processed.box.gro -p PROTEIN_processed.top -
o PROTEIN_processed.box.em.tpr -po em.out.mdp

mdrun_openmm -c PROTEIN_processed.box.em.gro -s
PROTEIN_processed.box.em.tpr
```

and finally followed by `grompp` and `mdrun_openmm` for the production MD run:

```
grompp -f PROTEIN_processed.box.em.md.mdp -c
PROTEIN_processed.box.em.gro -p PROTEIN_processed.top -o
PROTEIN_processed.box.em.md.tpr -po md.out.mdp

mdrun_openmm -s PROTEIN_processed.box.em.md.tpr -o
PROTEIN_processed.box.em.md.trr -c PROTEIN_processed.box.em.md.gro -cpo
PROTEIN_processed.box.em.md.cpt
```

Supplementary text 2. Results

Statistical significance of the K_d values.

The formal statistical significance of the differences between K_d values of the mutants and WT can only be tested for a few mutants, namely for those for which more than three measurements of K_d were performed using the same batch of IFN-γ-SC. We used the two-sample *t*-test comparing two normally distributed means [1]. The null hypothesis (H0) of the test is the identity of K_d values of the mutant and WT: $K_{d(mut)} – K_{d(WT)} = 0$, the alternative hypothesis (H1) is a higher affinity of the mutant, i.e. $K_{d(mut)} – K_d(WT) < 0$. The values of *t* are -38.1 for N96W, -1.6 for H222R, and +8.3 for the triple mutant, respectively. Because one-tailed value of the parameter *t* for ~15 degrees of freedom is ±1.75, the higher affinity of N96W is highly significant (-38.1 < -1.75) while the improvement of binding for H222R is insignificant (-1.6 > -1.75); H222R can indeed be called a neutral binder. The degrees of freedom were calculated as $N_{(mut)} + N_{(WT)} – 2$; the numbers of measurements, N, are in Table 3.

Variability of SPR data measured for different batches of IFN-γ-SC.

Table 3 reports SPR data determined with one batch of IFN-γ-SC. When the measurements were carried out with four different batches of IFN-γ-SC, the averages of K_d values remained in general agreement but the error margins of the latter measurements were much higher: K_d values for WT, N70G and N96W are 21±9 (13), 19±17 (8), and 5.3±3.5 (7) nM, respectively (confidence limits calculated at the 95% level, number of measurements in parentheses). Therefore, individual values of K_d from measurements with different batches of IFN-γ-SC, which do not allow statistical treatment, should not be directly compared. All IFN-γ-SC batches were prepared by the same protocol; large error margins are likely caused by hard-to-control proteolysis of the C-terminus during purification process that has been reported previously [2].

Dissociation constants of two variants can be used to calculate the relative changes of Gibbs energy of their interaction; for dissociation constants of WT $(K_d)_{WT}$ and a mutant $(K_d)_{mut}$:

$$\Delta\Delta G = –RT\ln(K_d)_{mut} - \{-RT\ln(K_d)_{WT}\} = –RT\ln\{(K_d)_{WT}/(K_d)_{mut}\}$$

Because of stability issues with IFN-γ-SC described above, these ΔΔG values need to be calculated from K_d measurements using the same batch of IFN-γ-SC.

Table 3 does not list k_{on}, k_{off}, and K_d of the "negative" mutants 15, 16, and 17 because their K_d values were measured only once using batch of IFN-γ-SC different from what was used for the other variants.

Table S1. Sequences used for the global alignment of the IFN-γ-Rx sequences from 19 various species. The alignment is shown in Figure 2. Listed are GenBank GI codes of all 32 sequences and names of the proteins.

1. gi|145975948 truncated interferon-gamma receptor 1 [Homo sapiens] human.
2. gi|4557880 interferon gamma receptor 1 precursor [Homo sapiens] human
3. gi|189069218 unnamed protein product [Homo sapiens] human
4. gi|62897165 interferon gamma receptor 1 variant [Homo sapiens] human.
5. gi|13562049 interferon-gamma receptor [Homo sapiens] human
6. gi|632543 interferon-gamma receptor alpha chain [Homo sapiens] human.
7. gi|90083401 unnamed protein product [Macaca fascicularis] crab-eating macaque, species, primates.
8. gi|297291656 PREDICTED: interferon gamma receptor 1-like isoform 1 [Macacamulatta] crab-eating macaque, species, primates.
9. gi|297291658 PREDICTED: interferon gamma receptor 1-like isoform 2 [Macacamulatta] crab-eating macaque, species, primates.
10.gi|197100085 interferon gamma receptor 1 [Pongo abelii] Sumatran orangutan, species, primates.
11.gi|332213427 PREDICTED: interferon gamma receptor 1 isoform 1 [Nomascus leucogenys] Northern white-cheeked gibbon, species, primates.
12.gi|114609481 PREDICTED: interferon gamma receptor 1 isoform 5 [Pan troglodytes] chimpanzee, species, primates.
13.gi|296483981 interferon gamma receptor 1 [Bos taurus] cattle, species, even-toed ungulates
14.gi|78050063 interferon gamma receptor 1 [Bos taurus] cattle, species, even-toed ungulates.
15.gi|45385782 interferon gamma receptor 1 [Bos taurus] cattle, species, even-toed ungulates.
16.gi|45385784 interferon gamma receptor 1 [Cervus elaphus] red deer, species, even-toed ungulates.
17.gi|295444941 interferon gamma receptor 1 [Sus scrofa] pig, species, even-toed ungulates.
18.gi|194216473 PREDICTED: similar to interferon gamma receptor 1 [Equus caballus] horse, species, odd-toed ungulates.
19.gi|74198189 unnamed protein product [Mus musculus] house mouse, species, rodents.
20.gi|6754306 interferon gamma receptor 1 precursor [Mus musculus] house mouse, species, rodents.
21.gi|309329 interferon-gamma receptor precursor [Mus musculus] house mouse, species, rodents.
22.gi|149039622 interferon gamma receptor 1 [Rattus norvegicus] Norway rat, species, rodents.
23.gi|38541396 Interferon gamma receptor 1 [Rattus norvegicus] Norway rat, species, rodents.
24.gi|16758624 interferon gamma receptor 1 [Rattus norvegicus] Norway rat, species, rodents.
25.gi|334324216 PREDICTED: interferon gamma receptor 1-like [Monodelphis domestica] gray short-tailed opossum, species, marsupials.
26.gi|57031680 PREDICTED: similar to Interferon-gamma receptor alpha chain precursor (IFN-gamma-R1) (CD119 antigen) (CDw119) [Canis familiaris] dog, subspecies, carnivores.
27.gi|281354680 hypothetical protein PANDA_003082 [Ailuropoda melanoleuca] giant panda, species, carnivores.
28.gi|224047948 PREDICTED: similar to interferon gamma receptor 1 [Taeniopygia guttata] zebra finch, species, birds.
29.gi|194332850 interferon gamma receptor 1 [Gallus gallus] chicken, species, birds.
30.gi|326915840 PREDICTED: interferon gamma receptor 1-like [Meleagris gallopavo] turkey, species, birds.
31.gi|118404146 interferon gamma receptor 1 [Xenopus (Silurana) tropicalis] western clawed frog, species, frogs & toads
32.gi|211956284 soluble IFN-g receptor [Deerpox virus W-1170-84] Deerpox virus.

Table S2. Mutagenesis primers designed for the introduction of single residue substitution into IFN-γ-Rx WT. Mutated nucleotides are underlined.

Mutant	Primers	Length
N65R	GTTTTTACCGTCGAAGTGAAACGTTATGGCGTGAAAAATAGCGA<br>TCGCTATTTTTCACGCCATAACGTTTCACTTCGACGGTAAAAAC	44 bp
N70G	GAAAAACTATGGCGTGAAAGGCAGCGAATGGATCGATGCG<br>CGCATCGATCCATTCGCTGCCTTTCACGCCATAGTTTTTC	40 bp
S95R	ATCATGTGGGCGACCCGCGTAACTCCCTGTGGGTT<br>AACCCACAGGGAGTTACGCGGGTCGCCCACATGAT	35 bp
N96F	CATGTGGGCGACCCGAGTTTCTCCCTGTGGGTTCGTGTC<br>GACACGAACCCACAGGGAGAAACTCGGGTCGCCCACATG	39 bp
N96W	GATCATGTGGGCGACCCGAGTTGGTCCCTGTGGGTTCGTGTCAA<br>TTGACACGAACCCACAGGGACCAACTCGGGTCGCCCACATGATC	44 bp
K115Y	GAAAGAATCAGCGTATGCCTACTCGGAAGAATTCGCCGTG<br>CACGGCGAATTCTTCCGAGTAGGCATACGCTGATTCTTTC	40 bp
T166M	ATGACCCGGAAACCATGTGTTACATTCGTG<br>CACGAATGTAACACATGGTTTCCGGGTCAT	30 bp
T166Y	GTCGATTATGACCCGGAAACCTATTGTTACATTCGTGTTTATAACG<br>CGTTATAAACACGAATGTAACAATAGGTTTCCGGGTCATAATCGAC	46 bp
H222R	TGAAGGCGTTCTGCGTGTCTGGGGTGTCA<br>TGACACCCCAGACACGCAGAACGCCTTCA	29 bp
Y66L	CCGTCGAAGTGAAAAACCTGGGCGTGAAAAATAGCG<br>CGCTATTTTTCACGCCCAGGTTTTTCACTTCGACGG	36 bp
S71E	GAAAAACTATGGCGTGAAAAATGAAGAATGGATCGATGCGTGCATC<br>GATGCACGCATCGATCCATTCTTCATTTTTCACGCCATAGTTTTTC	46 bp
H222D	CTGAAGGCGTTCTGGATGTCTGGGGTGTC<br>GACACCCCAGACATCCAGAACGCCTTCAG	29 bp

Table S3. Color-coded values of ΔΔG calculated using FoldX for chains C/ABD of the PDB structure 1fg9. Red indicates stabilization, blue destabilization. The first set of ΔΔG values estimates the influence of mutations on stability of the whole IFN-γ/IFN-γ-Rx complex (a), the second set of ΔΔG values estimates (de)stabilization of the interaction between the receptor molecule and the rest of the IFN-γ/IFN-γ-Rx complex (b).

a)

res	G	A	V	L	I	S	T	C	M	N	Q	K	R	H	P	D	E	F	Y	W
K_64	3.33	1.7	0.32	-	0.2	2.85	2.47	1.2	0.6	2.12	0.59	0.0	-	24.2	8.1	4.91	5.72	9.7	14.4	9.97
N_65	0.38	-	-	-	-	-	-	-	-	0.0	-	-	-	-0.44	-	0.76	0.84	-	-1.57	-
Y_66	2.90	1.6	1.77	1.12	1.6	2.41	3.13	1.5	-	1.83	2.54	0.22	0.24	1.37	3.4	3.41	4.56	-	0.00	-
G_67	0.0	3.0	4.22	1.51	3.0	4.25	5.46	3.4	3.2	3.32	2.66	2.65	5.04	3.61	9.5	2.46	3.74	2.7	3.10	4.23
V_68	2.22	0.6	0.0	-	-	1.66	1.31	0.8	1.0	0.38	1.15	1.59	1.20	3.73	6.0	2.54	1.14	4.1	5.38	5.19
K_69	0.08	-	0.17	0.02	0.1	-	-	0.0	0.3	0.36	0.15	0.0	-	0.35	-	-	0.05	0.0	0.05	0.13
N_70	-	-	0.19	-	-	-	0.29	-	-	0.0	-	-	-	-0.64	-	-	-	-	-0.62	-
S_71	0.47	1.1	1.51	0.54	1.8	0.0	0.98	0.5	0.9	0.07	0.61	0.38	0.73	1.07	0.8	0.98	0.94	0.2	0.32	0.21
E_72	-	-	0.31	0.24	0.1	-	-	0.0	0.6	0.40	0.19	0.05	0.26	0.32	-	0.25	0.0	-	-0.77	0.26
W_73	2.37	2.3	1.99	1.36	1.9	3.06	2.73	2.3	0.9	3.14	3.31	3.58	4.01	2.83	1.1	2.76	2.87	1.8	1.29	0.0
D_93	0.53	-	2.66	-	-	0.39	-	-	-	1.00	-	-	0.15	0.61	-	0.0	-	-	0.10	-
S_95	1.32	0.7	-	-	-	0.0	-	0.3	0.2	0.84	-	0.05	-	4.37	1.0	1.82	2.57	2.3	2.53	4.05
N_96	0.89	0.4	0.34	-	-	0.96	0.41	0.5	-	0.0	0.42	0.47	-	0.18	3.8	0.35	1.61	-	-0.21	-
S_97	-	-	-	-	-	0.0	-	-	-	-	-	-	1.68	-1.42	-	-	-	-	-2.84	-
W_99	2.90	1.7	0.00	0.63	-	2.15	1.07	1.4	0.5	2.54	1.52	2.06	3.52	2.24	3.0	3.35	3.24	0.3	1.21	0.0
K_115	0.94	0.6	0.15	-	-	0.65	0.35	0.3	-	0.73	0.23	0.0	1.05	0.86	0.3	0.83	0.39	-	-1.50	0.43
S_116	0.18	-	2.04	-	0.1	0.0	0.42	0.5	-	-	1.89	1.92	2.26	7.47	3.2	2.27	2.01	7.8	10.3	7.19
E_118	-	-	0.35	-	1.0	-	0.22	-	-	-	-	-	-	0.12	-	0.03	0.0	-	-0.90	-
R_123	2.44	1.3	2.30	0.92	2.5	2.39	2.46	1.6	1.6	1.62	2.27	1.11	0.0	2.01	0.7	4.48	4.24	1.2	0.97	2.46
E_164	-	0.2	2.00	0.26	2.1	0.38	1.42	0.0	0.2	-	-	-	0.12	-0.17	2.6	0.09	0.0	-	-0.11	0.08
T_165	0.85	0.1	0.35	-	-	0.00	0.0	0.3	-	1.22	0.56	-	-	1.00	2.5	3.63	2.40	2.2	1.71	6.09
T_166	-	-	-	-	-	-	0.0	-	-	-	-	-	0.26	0.17	-	0.88	1.15	-	-0.94	-
Y_168	-	-	-	0.34	0.2	-	-	-	0.4	-	0.71	-	0.29	0.11	2.0	2.67	2.49	-	0.00	0.17
R/F_17	-	-	-	-	-	0.29	-	-	-	0.45	-	-	0.0	-1.28	2.0	-	-	-	-2.47	-
V_171	2.42	1.1	0.0	0.69	0.0	1.76	1.33	0.8	1.2	1.18	0.57	0.71	1.17	1.67	0.8	1.65	1.13	0.7	1.89	1.20
K_186	0.29	0.3	0.64	-	0.3	0.04	0.58	-	-	0.02	0.20	0.0	0.45	0.64	-	-	-	-	0.21	0.07
T_189	0.92	-	-	-	0.4	0.33	0.0	0.6	-	0.84	0.00	0.47	0.44	1.78	1.7	2.07	1.51	0.4	0.71	0.90
Q_190	-	-	-	-	-	-	-	-	-	-	0.0	0.40	0.92	0.23	0.3	-	0.49	-	-0.60	-
K_191	0.60	0.0	0.73	-	0.3	0.49	0.73	0.6	-	0.00	-	0.0	0.33	0.52	-	0.00	-	0.5	0.39	0.02
E_192	-	-	0.02	-	0.6	-	-	-	0.6	-	0.35	0.76	1.41	0.56	5.8	-	0.0	0.2	2.37	2.60
D_193	-	-	0.28	-	0.2	-	0.09	-	0.0	-	-	-	-	-0.39	-	0.0	-	-	-0.45	-
E_197	-	-	0.02	-	-	-	0.17	0.1	-	0.12	-	-	-	0.40	-	0.33	0.0	-	-0.45	0.12
V_220	2.56	1.4	0.0	0.10	-	2.26	1.61	1.4	1.0	2.49	1.66	1.86	2.10	1.92	4.0	2.22	1.11	0.5	0.94	1.09
L_221	3.13	2.6	2.70	0.0	2.6	2.13	2.02	2.2	1.7	3.20	3.15	2.47	2.90	1.68	3.5	4.78	4.11	2.2	8.35	3.80
H_222	-	-	0.22	-	0.1	-	0.13	-	-	-	-	-	-	0.00	-	-	-	-	-1.69	-
V_223	1.29	2.0	0.0	-	-	2.42	1.21	0.8	-	1.55	0.83	0.14	2.10	34.5	3.4	2.30	2.27	3.8	5.30	5.32
W_224	3.33	3.0	2.90	1.91	2.9	3.81	4.23	3.0	2.2	4.20	3.54	2.82	3.10	2.42	2.2	4.52	4.45	1.0	1.53	0.0
G_225	0.0	1.8	6.50	4.89	8.0	2.78	5.98	3.0	4.1	2.94	5.28	3.55	4.48	5.09	7.4	4.21	5.79	4.4	5.26	5.03
V_226	1.26	0.4	0.0	-	-	0.65	0.98	0.3	0.0	0.79	0.80	0.42	0.49	1.94	-	1.48	0.94	0.0	0.41	1.34
T_227	0.13	-	-	-	-	-	0.0	-	-	-	-	-	-	-0.12	-	0.70	-	-	-0.94	-

Table S3 b)

res	G	A	V	L	I	S	T	C	M	N	Q	K	R	H	P	D	E	F	Y	W
K_64	1.68	1.7	1.22	1.15	1.3	1.66	1.93	1.2	1.2	1.76	0.53	0.0	-	0.96	1.6	3.31	3.75	3.0	3.80	2.79
N_65	0.29	0.2	-	-	-	0.20	-	0.0	-	0.0	-	-	-	0.00	0.1	0.11	0.09	0.0	0.01	0.03
Y_66	4.20	3.5	2.86	2.85	4.1	4.30	4.20	3.5	1.1	4.46	3.61	1.83	1.16	2.52	3.5	4.73	4.24	-	0.0	1.16
G_67	0.0	0.9	0.14	-	-	1.24	1.22	0.7	-	0.96	-	0.31	1.20	0.74	2.0	-	0.41	0.1	0.23	0.90
V_68	1.92	0.7	0.0	1.97	0.5	0.91	1.68	0.3	2.0	-	-	1.11	0.97	1.86	5.2	0.50	0.04	0.3	2.04	2.18
K_69	1.40	1.4	1.45	1.39	1.4	1.42	1.44	1.4	1.4	1.45	1.41	0.0	1.46	1.43	1.1	1.19	1.44	1.3	1.37	1.37
N_70	0.02	-	0.21	0.18	0.2	-	0.15	0.0	0.1	0.0	0.11	0.08	0.03	0.04	-	0.29	0.17	0.0	0.07	0.14
S_71	2.21	2.2	1.96	1.49	1.5	0.0	0.18	1.8	1.5	1.62	1.70	1.30	1.17	1.99	1.8	2.19	2.09	1.4	1.69	1.52
E_72	-	-	-	-	-	-	-	-	-	-	-	-	-	-	-	-	0.0	-	-	-
W_73	0.25	0.1	-	0.18	0.0	0.10	-	0.1	0.1	-	0.29	-	0.01	-	-	0.08	0.07	-	0.40	0.0
D_93	0.05	-	-	-	-	0.09	0.07	-	-	0.10	0.06	0.07	0.27	0.35	-	0.0	-	0.2	0.29	0.19
S_95	0.51	0.2	0.10	-	-	0.0	0.22	0.2	-	0.19	0.12	-	-	0.33	0.4	0.39	0.98	0.1	0.29	-
N_96	-	-	-	-	-	-	-	-	-	0.0	-	-	-	-	-	0.04	0.22	-	-	-
S_97	-	0.2	-	0.04	-	0.00	0.32	-	-	-	0.20	-	0.0	-	-	-	-	-	-	-
W_99	0.63	0.4	0.29	0.24	-	0.62	0.50	0.2	-	0.74	1.09	-	1.89	0.72	0.3	-	0.97	0.6	-	0.0
K_115	0.04	0.1	0.17	0.21	0.1	0.23	0.10	0.2	0.2	0.48	0.01	0.0	-	0.15	0.0	0.18	0.40	0.2	0.22	0.23
S_116	-	-	-	-	0.0	0.0	-	-	-	-	-	-	-	-	-	-	-	-	-	-
E_118	-	-	-	-	-	-	-	-	-	-	-	-	-	-	-	-	0.0	-	-	-
R_123	1.40	1.1	1.32	1.60	1.5	1.26	1.27	1.2	1.9	1.58	2.20	0.94	0.0	1.29	0.9	2.20	2.47	1.1	0.93	1.41
E_164	0.30	0.3	0.62	0.47	0.4	0.35	0.39	0.3	0.2	0.26	0.26	0.29	0.34	0.33	0.3	0.16	0.0	0.4	0.45	0.46
T_165	0.03	0.0	0.15	0.07	0.1	0.02	0.0	0.0	0.0	0.11	0.06	0.05	0.13	0.19	0.0	0.29	0.10	0.3	0.28	0.02
T_166	0.43	0.5	-	-	-	0.01	0.0	-	-	-	-	0.22	0.09	0.67	0.1	1.98	1.43	0.1	-	-
Y_168	0.14	0.0	-	-	-	0.09	-	0.0	-	0.12	-	-	-	-	-	0.07	-	0.1	0.0	-
R/F_17	0.30	0.2	0.30	0.29	0.3	0.29	0.30	0.3	0.2	-	0.56	0.13	0.0	0.29	0.3	0.41	0.50	0.2	0.27	0.27
V_171	-	-	0.0	0.00	0.0	-	-	-	-	-	0.31	-	-	-	-	-	-	-	-	0.08
K_186	-	-	-	-	-	-	-	-	-	-	-	0.0	0.04	-	-	-	-	0.0	0.04	0.10
T_189	0.00	-	0.00	0.00	0.0	0.00	0.0	0.0	0.0	0.00	0.00	0.02	0.08	0.01	0.0	-	-	0.0	0.10	-
Q_190	-	-	-	-	-	-	-	-	-	-	0.0	-	-	-	-	-	-	0.1	0.09	0.45
K_191	-	-	0.11	0.17	0.0	-	0.13	-	-	-	0.06	0.0	0.01	-	0.0	-	0.05	0.1	-	0.46
E_192	0.04	0.0	0.04	0.04	0.0	0.04	0.04	0.0	0.0	0.04	0.04	0.04	0.04	0.04	0.1	-	0.0	0.0	0.07	0.03
D_193	0.06	0.0	0.06	0.06	0.0	0.06	0.06	0.0	0.0	0.06	0.06	0.08	0.12	0.10	0.0	0.0	0.01	0.0	0.06	0.06
E_197	0.10	0.0	0.10	0.10	0.1	0.10	0.13	0.1	0.1	0.09	0.10	0.11	0.14	0.09	0.1	-	0.0	0.0	0.09	0.09
V_220	-	-	0.0	0.01	0.0	-	0.03	-	0.1	0.39	0.12	0.46	0.76	0.19	-	-	-	0.3	0.37	-
L_221	-	0.0	0.12	0.0	0.0	0.05	0.04	-	-	-	-	-	-	-	0.3	-	-	-	-	-
H_222	-	0.0	-	-	-	0.00	-	-	-	-	-	-	-	0.0	-	0.04	-	-	-	-
V_223	1.62	1.2	0.0	0.49	0.0	0.76	1.05	0.3	-	1.58	1.03	0.42	0.58	2.89	1.7	2.88	2.34	4.6	6.32	5.13
W_224	1.61	1.8	1.82	1.15	1.5	1.99	1.86	1.8	0.7	2.53	2.04	1.66	1.60	1.11	1.7	2.02	2.84	1.0	1.27	0.0
G_225	0.0	0.1	2.31	2.00	4.7	0.58	2.48	1.3	1.5	0.94	2.42	1.10	0.35	2.60	1.3	1.54	2.60	2.2	2.45	2.21
V_226	0.00	0.0	0.0	-	-	0.00	0.00	0.0	0.0	-	-	-	-	0.00	0.1	0.02	-	-	-	-
T_227	0.00	0.0	0.01	0.12	0.0	0.00	0.0	0.0	0.1	0.01	-	0.04	0.06	0.10	0.0	-	-	0.2	0.42	0.17

Table S4. Preliminary data of temperature dependency of affinities between IFN-γ-Rx and IFN-γ-SC for selected mutants measured by SPR at 15 °C, 20 °C, 25 °C, 30 °C, and 35 °C.

Mutant		K_d [nM]					ΔH (*)	ΔS (*)	R^2
ID	mutant	15 °C	20 °C	25 °C	30 °C	35 °C	[kJ/mol]	[J/molK]	(**)
2	N70G	14.7	17.3	23.6	49.2	77.0	0.93	-1.03	0.94
3	S95R	38.6	44.4	69.4	107.	153.	0.78	-0.62	0.97
5	N96W	5.55	5.28	6.1	12.2	24.3	0.80	-0.46	0.81
9	H222R	16.4	19.4	30.1	56.0	103.	1.01	-1.32	0.95
WT	--	16.8	21.7	30.5	59.9	92.8	0.94	-1.11	0.97

(*) Enthalpic and entropic contributions to free energy (values of ΔH and ΔS, respectively) were calculated from equation $\Delta G = \Delta H - T\Delta S$ by fitting the linear equation $\ln K_d = \{-\Delta H/R\}/T + \Delta S/R$ for coefficients $-\Delta H/R$ and $\Delta S/R$ assuming that ΔH and ΔS are temperature independent. Values at 35 °C were not considered for the determination of the temperature dependencies of ΔH and ΔS.

(**) Correlation coefficient of the least square fit of the regression equation mention above.

Figure S1. Melting temperatures of the WT and selected mutants. Curves obtained from fluorescence-based thermal shift assay of IFN-γ-Rx WT (blue, melting temperature 55 °C), N96W (red, melting temperature 48 °C) and N96W+H222R (green, melting temperature 47 °C). a) Normalized data of PBS buffer subtracted data of IFN-γ-R WT, N96W and N96W+H222R. b) First derivative of the data represented in (a). The lowest value of each curve represents melting temperature (Tm) of IFN-γ-R variants.

a)

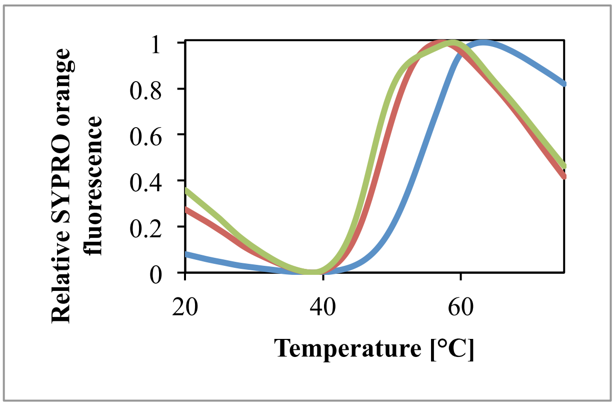

b)

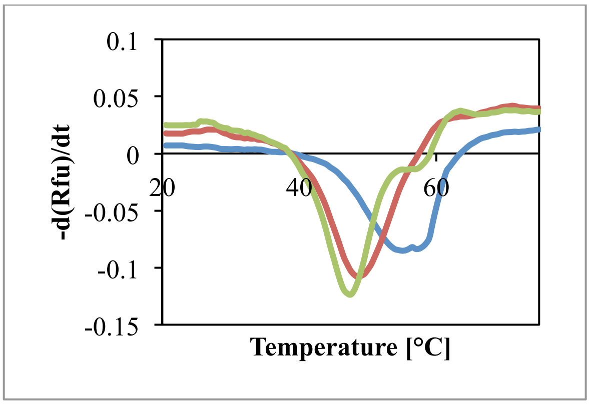

Supplement References

1. Freund JE & Williams FJ (1966) Dictionary/outline of basic statistics. New York: McGraw-Hill.

2. Landar A, Curry B, Parker MH, DiGiacomo R, Indelicato SR, *et al.* (2000) Design, characterization, and structure of a biologically active single-chain mutant of human ifn-gamma. ***J Mol Biol*** **299**: 169-179.
